# A protocol study of participatory action research: integrated care pathway for pregnant women with heart disease in Indonesia

**DOI:** 10.1186/s12913-020-05769-3

**Published:** 2020-10-09

**Authors:** Suryani Yuliyanti, Adi Utarini, Laksono Trisnantoro

**Affiliations:** 1 Public Health Department, Faculty of Medicine, Universitas Islam Sultan Agung, Jl. Kaligawe Raya km 4, Semarang, 50122 Indonesia; 2 Doctoral Study Programme, Faculty of Medicine, Public Health and Nursing, Universitas Gadjah Mada, Jl. Farmako Sekip Utara, Yogyakarta, Sinduadi, Mlati, Special Region of Yogyakarta 55281 Indonesia; 3 Department of Health Policy and Management, Faculty of Medicine, Public Health and Nursing, Universitas Gadjah Mada, Jl. Farmako Sekip Utara, Yogyakarta, Sinduadi, Mlati, Special Region of Yogyakarta 55281 Indonesia

## Abstract

**Background:**

Heart diseases are increasingly identified as an important indirect cause of maternal mortality in several cities in Indonesia. The management of pregnancy with heart diseases requires a multidisciplinary approach, and interprofessional collaboration practice (IPCP) is critical to improving the quality of patient care. To enable the effective implementation of IPCP, integrated care pathways (ICPs) are needed to define the roles and responsibilities of the health professionals involved. This study aims to examine the obstacles and enabling factors of IPCP, to develop and use ICPs in the implementation of IPCP in health care services for pregnant women with heart diseases.

**Methods:**

A participatory action study consisting of four stages (diagnostic, planning, implementation, and evaluation) will take approximately 2 years after consensus of ICPs are made. The primary data collection process will employ consensus, observations, focus group discussions, and in-depth interviews throughout the four stages, while secondary data from referral documents and medical records will be collected mainly during the diagnostic and evaluation stages. The findings are being analysed and will then be used to develop an ICPs through consensus building at the planning stage to be applied in the implementation stage. Finally, the implementation outcome, including acceptability, adoption, appropriateness, and feasibility of IPCP, will be assessed in the evaluation stage. All qualitative data will be analysed thematically by two coders using NVIVO 12 software.

**Discussion:**

This research aims to assess the needs of IPCP, develop and use the ICPs in the implementation of IPCP in health care services for pregnant women with heart diseases. Findings from this study will be used for health service planning and policy making to strengthen practice of IPCP during the referral process. As a result, pregnant women with heart disease will have better access to high-quality services at every health care facility to reduce maternal mortality.

**Trial registration:**

Retrospectively registered in the ISRCTN registry with study ID ISRCTN82300061 on Feb 6, 2019.

## Background

Cardiac diseases rank first in the most common indirect cause of maternal death in developed countries [1–5]. They are, although not the leading cause, are the important indirect cause of maternal mortality in developing countries. Due to the limited diagnostic modalities and poor management, the true number of maternal cardiac cases is likely far greater than the number reported [1, 2, 6, 7]. In Indonesia, maternal deaths caused by heart diseases also have been increasing for the last four preceding years [8–11], in parallel with a general increase of non-communicable diseases as a cause of maternal death [12]. There have been efforts [13] to address the maternal referral delays [14]. However, these are not specifically aimed to address the health service quality (third phase delay) which has been reported as the most common cause of maternal death [14–16]. Due to its complexity, cardiovascular disease in pregnancy requires an integrated care [17, 18], the highest degree of Interprofessional Collaboration Practice (IPCP) during every phase of pregnancy, from prenatal counselling, antenatal care, delivery, to postnatal management [19, 20]. Better IPCP will help improve the quality of health services [21] by preventing clinical mismanagement [14].

IPCP has long been studied elsewhere and studies have demonstrated that successful implementation of IPCP can lead to improved quality of health care [21], particularly through enhancing patient safety and satisfaction of both the patients and health care professionals [20, 22–24]. For illustration, a study in Germany found that an interprofessional share-decision-making training model has significantly increased satisfaction of health professionals [25]. While a study in the UK assessing interprofessional communication among the delivery team concluded that interprofessional tensions, workload stresses and the design of the environment can restrict communication, with implications for safety [23]. IPCP has been proven to improve satisfaction for women who receive care during pregnancy, delivery to postpartum based on a study in Australia [26].

IPCP occurs when different health professionals working together in order to provide high quality of care [27]. The practice can be more challenging, yet needed, for patients with complex health problems. Mulvale et al. developed a gearing up theory to explain interrelated factors contributing to IPCP, i.e. individual, micro-gear (team), meso-gear (organizational) and macro-gear (policy). Among these factors, team factors are the major factor for successful implementation of IPCP [28]. This addresses the core issues arising when different professionals work together. Power dynamics, poor communication patterns, lack of understanding and clarity of roles and responsibilities are most common barriers in the process of IPCP [29–31]. The organizational factors, i.e. barriers of intra and inter-organizational collaboration, workloads, roster, availability of senior consultants, availability of needed equipment and products, and lack of funding, have been identified as the leading contributory factors to indirect maternal death [28, 32]. Auschra et al. also highlighted the importance to address the organizational barrier to establish an integrated health service delivery [33].

One known approach to enhance interprofessional and inter-organizational relationships is through integrated care pathways (ICPs) [34]. ICPs are locally agreed structured multidisciplinary care plans which detail critical steps in the care of patients with a specific clinical problem. Ideally, it is arranged by a multidisciplinary agreement to accommodate all health professional on the appropriate position based on their roles and responsibilities [35]. Even though there has not been a conclusive evidence on correlation between ICPs and IPCP [36, 37], several studies demonstrated that a clearly defined role and responsibility of health professionals in ICPs promotes an effective implementation of IPCP [19, 38–41].

We are aware that the guidelines of a multidisciplinary approach for pregnant women with cardiac diseases have been established and implemented in several developed countries, such as in the UK [42, 43], Japan [44], USA [45] and Australia [46]. However, our literature search on Pubmed found limited evidence of their effective implementation in the respective countries. One study in England merely showed the barriers in implementing the models [19]. The study concluded that different interest and expertise lead to various model of care pathway for pregnant women with cardiac disease called “fragmented care”. To address this issue, the study suggested that a further study on “joined care” is needed. This study protocol is a type of “joined care”, defined as an “interdisciplinary team approach guided by consensus building, mutual respect, and a shared vision of health care that permits each practitioner and the patient to contribute their particular knowledge and skills within the context of a shared, synergistically charged plan of care” [19, 47].

The study aims to examine the obstacles and enabling factors of IPCP, to develop and use ICPs in the implementation of IPCP in health care services for pregnant women with heart diseases. The specific objectives are to:

1. Identify obstacles and enabling factors of current practice of IPCP,
2. Develop ICPs in the referral process from primary to tertiary care facilities for pregnant women with heart diseases,
3. Integrate the use of ICPs in the implementation of IPCP, and
4. Evaluate the implementation outcomes of IPCP.

## Methods

**Fig. 1:**
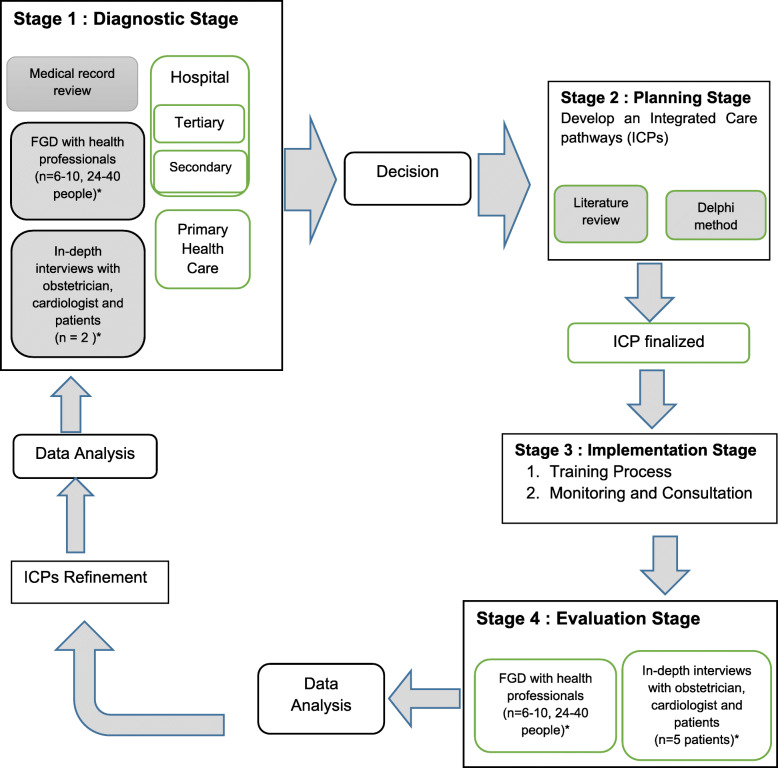
Project Flowchart. Note: The above flowchart shows the minimum number of participants required (indicated by *). Cycles of PAR will continue until sufficient understanding to progress to the next cycle is achieved

Participatory Action Research (PAR) is a systematic, rigorous and emergent research process in which behavioural science knowledge is applied and integrated to improve understanding of practical aspects and of practice itself and solve real organisational problems [48, 49]. The main focus of PAR is the participants’ involvement in the effort to improve their own practices [50]. Four stages of PAR will be conducted (Fig. 1). The first stage will use in-depth interviews and Focus Group Discussions (FGDs) to understand the enabling and hindering factors of IPCP. Stage two will employ literature review and Delphi method to develop ICPs for pregnant women with heart diseases, followed by implementation of ICPs in stage three. Finally, the proposed model of ICPs will be evaluated in stage four. This study will take approximately 2 years after consensus of ICPs is made [51, 52]. Three groups of participants will be involved throughout this study, i.e. midwives who will act as co-researchers in their respective workplace, health professionals, and representatives from relevant organisations, such as the municipality health office and branch of the Indonesian Social Insurance Administration Organization (or known as Badan Penyelenggara Jaminan Kesehatan/BPJS) at the city level. Data collection will be held face to face in their workplace; while for the patients, the interviews will be carried out in their home. Details for the data collection and analysis are described below for each stage in PAR. The reporting of this study will be referring to the COREQ statement [53] and guideline for best practices in the reporting of PAR from Smith et al. [54].

### Study setting

Central Java is a province located in Java Island with a population of more than 34 million. The number of maternal deaths in Central Java recently decreased from 711 (2014) to 609 (2016), with maternal mortality rates of 126 (2014) and 109 (2016) per 100,000 live births. The capital city, Semarang, hosts the referral centre and the most advanced health care facilities in the province. Seven Comprehensive Emergency Obstetric and Neonatal care or CEmONC hospitals and seven Basic Emergency Obstetric and Neonatal Care or BEmONC primary health care centres provide services for a population of 1.65 million in Semarang. Each year, more than 25,000 deliveries occur, and in 2017, the maternal mortality ratio (MMR) was 88 per 100,000 live births. Despite the decrease in the MMR over the last three preceding years, the total number of maternal deaths due to heart diseases have increased from two out of 35 deaths in 2015 to five out of 23 deaths in 2017 [8].

The Sultan Agung Islamic (SAI) hospital is a tertiary private teaching hospital in Semarang with more than 1000 deliveries annually. This CEmONC hospital provides comprehensive emergency obstetric and neonatal care delivered by eight obstetricians (consisting of two full-time and six part-time obstetricians), six paediatricians (consisting of one full-time and five part-time paediatricians), 15 nurses and 25 midwives. The facilities dedicated to obstetric care services consist of three emergency room beds dedicated to obstetric emergency cases, one ward with 20 beds for inpatient care, an outpatient service, one delivery room and two rooms for critical obstetric patients in the Intensive Care Unit (ICU).

The study will take place at tertiary hospital and its referral network, which includes three primary health care facilities and one secondary health care facility, as described in Table 1. The hospital was purposively chosen based on the number of pregnant women with heart disease.

**Table 1:** Characteristic of the health care facilities

Characteristic	Tertiary Hospital: SAI	Secondary Hospital: DRP	Primary health care facilities		
			PHC A	PHC B	PHC C
**Number of human resources**					
Midwives	25	6	6	4	4
Nurses	15	5	6	4	4
Physicians	15	10	5	4	4
Obstetricians	8	5	0	0	0
Cardiologists	7	0	0	0	0
**Services**					
BEmONC care	NA	NA	A	NA	NA
CEmNOC care	A	NA	NA	NA	NA
Outpatient	A	A	A	A	A
Inpatient	A	A	A	A	NA
ICU	A	A	NA	NA	NA

*SAI* Sultan Agung Islamic, *DRP* Demak Regional Public, *A* available, *NA* Not Available, *PHC* Public Health Centre

The Demak Regional Public (DRP) hospital is a secondary public hospital located 3.4 km from SAI hospital with 700 deliveries annually. This CEmNOC hospital provides a comprehensive emergency obstetric and neonatal care delivered by five obstetricians (two full-timers and one part-time obstetrician), and three paediatricians (two full-timer paediatricians and one part-time paediatrician), five nurses and six midwives. The facilities dedicated for obstetric care services consist of one emergency room bed, one ward with 20 beds for inpatient care, an outpatient service, one delivery room and one room for critical obstetric patients in the ICU.

### Stage 1: diagnostic stage

To understand the local context, this study begins with assessing the facilitating and hindering factors of IPCP in SAI hospital and its maternal referral network. In addition, the assessment is intended to provide an accurate description of current maternal referral patterns. The information will be obtained from health professionals and patients through in-depth interviews, FGDs, and review of medical records.

#### Participants

Health professionals providing services for pregnant women with heart diseases will be purposively selected for in-depth interviews and FGDs based on their role and knowledge of the existing maternal referral system [55]. For the interview, attempts will be made to obtain a maximum variation of participants in respect to demographic factors including gender, age and formal educational background; IPCP experiences and previous training [55]. Nine interviews will be conducted, i.e. with the heads of obstetrics and gynaecology and cardiology department in two hospitals and person in charge (PIC) in each five health care facilities in the study [56]. Four FGDs will be conducted, i.e. with hospital physicians, primary care physicians, hospital-primary care midwives and hospital nurses. Permanent employee with a minimum of one-year working experience will be purposively selected and approached by the PICs as FGD participants. Each group will consist of 6–10 participants.

Patients from every health care facility will be purposively selected for an in-depth interview regarding their health service experiences and perceptions toward the need for IPCP [55]. Since the number of pregnant women with cardiac diseases is limited, we, therefore, will purposively select patients who are pregnant with heart diseases and or have a history of heart diseases during pregnancy within 1 year (prior to the stage one) and have received medical service started from primary to tertiary health facilities.

#### Data collection

The interview and FGD data collection guides will be developed using the “gearing up” model by Mulvale [28] to identify the promoting and hindering factors of IPCP. The referral pattern will be assessed based on demographic data of the patient (patient’s age and address), obstetric complication, and mode of delivery [57]. Semi-structured interviews and FGD have been chosen to make sure all the necessary information are collected [56]. The scope of questions for the interviews and FGDs will cover input, process, promoting and barriers factors, knowledge, attitude, perception and experiences of both the existing IPCP and maternal referral process. Pilot testing of the guidelines will be carried out with physicians and midwives working in a different primary care setting. The first author (SY) will conduct the interviews. She will also facilitate the FGDs as moderator, accompanied by a research assistant (RA) [29, 56]. The interview will take between 30 and 90 min, while the FGDs will run for 60–90 min. Field notes during data collection and transcripts of recorded interviews and FGDs will be produced after each session. Repeat interviews will only be conducted if information from the previous interview is incomplete due to time constraints of the health professionals.

For the purpose of triangulation regarding IPCP and maternal referral services, a review of medical records of all pregnancies with heart diseases in the last 1 year at SAI hospital will be carried out. The following data will be extracted from medical records: diagnosis made by the referring and destination centers and degree of collaboration based on the integrated patient notes (number of case conferences discussing the patients, mode of communication among health professional). The medical records will be selected using the ICD-10 code of 0.99.4 for all deliveries (diseases of the circulatory system complicating pregnancy, childbirth and puerperium), Q.24.0–9 (congenital heart malformation), 0.90.3 (cardiomyopathy) and I00-I99 (heart diseases).

#### Analysis

A thematic analysis approach to data analysis will be used, involving coding and categorising. Theming refers to the drawing together of codes from interview and FGD transcripts to present the findings in a coherent and meaningful way. The six phases of thematic analysis pinpointed by Braun and Clarke 2006 will be applied [58]. First coding will be carried out by two coders (SY and RA). Codes will be grouped into subcategories and arranged into key categories using a process known as ‘describe-compare-relate’. These codes and categories will be further discussed with the second author (AU). The newly categorised data will be analysed in a subsequent session with participants to review categories for consistency and to identify key categories. Data saturation will be reached when no new information is sought and this will be discussed and agreed by the first author and research assistant. When there is a disagreement, consultation will be made to LT and AU as supervisors. The findings will be presented as coding tree, describing the codings, categories, sub-themes, and themes. Quotations will be presented to illustrate the categories and themes. The result will also present any diverse case and minor findings found in this study. The data will be analysed using NVIVO 12 software.

### Stage 2: planning stage

Stage two will focus on developing the ICPs draft through two activities: review of ICPs for pregnant women with heart disease and consensus to gain a local context of ICPs [59].

#### Review of sources

References on ICPs for pregnant women with heart diseases available in both Indonesian and English languages will be reviewed. As mentioned in the previous stage, the ICPs will be used to facilitate implementation of IPCP. Therefore the content of ICPs will consist of care processes for pregnant women with heart diseases from prenatal, antenatal, delivery, and postpartum period, as well as role and responsibility of each health professional, health care facilities involved, and cost of care. An online search will be conducted using the keywords that are relevant to pregnancy with heart diseases and maternity care pathway in combination using the Boolean operators ‘AND’ and ‘OR’ via Pubmed, web of science, Scopus and Google scholar (Table 2). To find the Indonesian resources, the keywords will be translated and will also be searched from the related health professional organization websites, i.e. the Indonesian Heart Association and Indonesian Obstetric and Gynaecology Association. This process aims to identify best practice for ICPs to develop a well-design model [60, 61]

**Table 2:** Keywords for search electronic database

Integrated care pathway	Pregnancy with heart diseases
Collaborative care pathway	Pregnancy with cardiac diseases
Integrated management	Pregnancy with cardiac disorder
Interprofessional Collaboration	Pregnancy with heart disorder
Guideline	

#### Consensus process

This study will apply two cycles of the Delphi method to achieve locally agreed ICPs. The findings from the review will be arranged by the PI to formulate the ICPs draft and will be consulted to expert and representatives from the relevant organization in the first cycle of Delphi method. The draft will then be discussed with health professional representative from each health care facilities to allow local context [62]. This process will be audio-recorded and observed by PI or RA using a modified McMaster Ottawa Scale to simplify observation in clinical setting. The scale covers examination of the collaboration aspect, including collaborative communication, share leadership, conflict resolution, team functioning, role and responsibility of each participant [63]. An interesting finding regarding the IPCP aspect during the process will be explored through subsequent individual interviews with the selected participants.

The second cycle of the Delphi method will provide participants with an opportunity to give further feedback on the model. Data will be clustered and confirmed with participants to ensure fairly representative data. Repetition of the method will continue until 80% of participants agreed on the model [61, 64].

#### Participants

Senior health professionals recruited in stage one with a structural position in their respective workplace alongside the liaison officers who are responsible for maternal referrals will be involved in making the consensus on the model. The representative of municipality health office will be asked to provide feedback on the appropriateness of the model with regards to the policy and existing resources available. Experts are senior community obstetrician acting as consultants of the maternal mortality reduction programme and senior cardiologist in tertiary hospital who will be invited to give feedback on the clinical aspects within the proposed model. The representative of the Indonesian Social Insurance Administration Organization/BPJS will be invited to provide feedback on the funding scheme of the model. Health professionals representative from each workplace will be then involved in the following stage of Delphi cycle [55].

#### Analysis

Data gathered in the first Delphi cycle will be qualitative in nature and will be analysed using the six-phase thematic analysis techniques. The modified McMaster Ottawa scale and the progress of agreement in the second cycle will be analysed descriptively and confirmed with the qualitative data.

### Stage 3: implementation stage

Two activities will be conducted to strengthen the support system in the implementation of ICPs models in this stage (i.e. training, ongoing monitoring and consultation) [65].

#### Training process

Training of the agreed ICPs from Stage 2 will be delivered to all health professionals in the study sites who are involved in managing pregnant women with heart diseases, and also attended by representatives from the municipality health office. Experts in the area of IPCP and content of ICPs will be asked to deliver the training. A one-day training will be organized in each health facility, to cover the following topics: an overview of IPCP and ICPs, the importance of IPCP for managing complex diseases, the content of ICPs and a more detailed explanation on implementation of ICPs.

#### Ongoing monitoring and consultation

After training has been completed, implementation of ICPs to strengthen IPCP will begin as pregnant women with heart diseases visit the health care facilities included in the study. Ongoing monitoring and consultation to the local health facilities will be continuously conducted by the Principal Investigator (PI) and co-researchers to document the process, identifying problems and finding the best solutions.

#### Participants

All health professionals, representative of the municipality health office, co-researchers and clinical consultants who contribute in designing the model will be involved in training activity, while the ongoing monitoring will only involve health professionals who implement the ICPs and co-researchers in their health facilities. The PI and co-researchers will provide ongoing monitoring and consultation throughout the implementation of ICPs.

#### Data collection

IPCP quizzes will be completed by all participants before and after the training to assess their knowledge improvement, while attitude and perception of IPCP will only be assessed after the training, using the Jefferson Scale of Attitudes Toward Interprofessional Collaboration (JeffSATIC) [66] and the Perception of Interprofessional Collaboration Model Questionnaire (PINCOM-Q) [67]. The ICP training process will be documented by RA to describe the participants’ responses. The following information regarding progress of implementation will be monitored and recorded: date of the first patient-reported to PI or co-researchers, implementation of IPCP, consultation request, and clinical meeting [65]. A modified McMaster Ottawa Scale [63] will be used by the co-researchers to observe IPCP in delivering care to pregnant women with heart diseases. In this stage the total number of pregnant women with cardiac disease will be recorded including detailed information regarding their identity, diagnosis, and level of facilities used during their prenatal, antenatal, and post-natal care. Observations will be conducted for every pregnant woman with heart diseases in the study setting to evaluate the intended and unintended outcome of the ICPs, to determine the progress made and learning from the actions using participatory tools such as progress markers, photos, and videos [68–70]. The intended outcomes include improvement of interprofessional communication, and patient outcomes such as length of stay and readmission [68, 69], whereas the unintended outcomes consist of interprofessional knowledge gaps, tension, and disrupted positive communication [70]. All health professionals and patients will be asked to provide consent before being photographed or videotaped.

#### Analysis

The score of IPCP quizzes will be compared using a paired t-test to determine training effectiveness. The attitude, perception and implementation toward IPCP will be analysed descriptively and classified based on their profession and workplaces. All qualitative data from observations dan field notes will be gathered and analysed with the data from subsequent stage.

### Stage 4: evaluation stage

This stage will focus on the formal evaluation after the three previous stages have been completed.

#### Participants

The participants in this stage will be to those health professionals and patients who participate in the ongoing monitoring activity during stage three.

#### Data collection

Interviews and FGDs will be conducted to assess both on being involved in a study using PAR (such as degree of participation and learning gained on ICPs and IPCP throughout the study) and the IPCP implementation outcome such as acceptability, appropriateness, adoption, feasibility, and implementation cost [71–73]. The interviews will be conducted with obstetricians, cardiologists and patients. For the FGD, four FGDs will be conducted with physicians working in primary care and hospitals, as well as midwives in all types of health facilities and hospital nurses. Each group will consist of 6–10 participants. All four stages will be applied to 5 patients for each cycle and will be repeated until four completed cycle of PAR.

#### Analysis

All data collected in this stage will be analyzed mirroring the analysis in stage one.

A summary of data collection in all four stages is illustrated in Table 3.

**Table 3:** Data collection activities in each stage of the study

Stages	Outcome	Data collection activities	Instrument	Variable	Participant
**Diagnostic stage**					
Determine local context of existing IPCP in maternal referral services for pregnancy with heart diseases	Policy factors Organizational factors Team factors Individual factors	Medical record review (obstetric cases in 2017)	Collecting document form	Existing referral system and IPCP	Medical record
		In-depth interviews and Focus group discussions (FGDs)	Interview and FGD guideline Audio recording	Contributing factor in IPCP implementation	Heads of obstetrics and gynaecology, and cardiology Person in charge (PIC) of maternal referral from each health care facility. Patients
**Planning stage**					
ICPs development	Agreed ICPs	Review of source	–		Principal investigator
		Consensus: Delphi method	JEFFSATIC PINCOM-Q Modified McMaster Ottawa Scale Audio recording	Attitude and Perception of IPCP on developing ICPs	Senior community obstetrician and cardiologist. The liaison officers The representatives of municipality health office. The representatives of the BPJS Senior health professionals
**Implementation stage**					
IPCP implementation	Description of IPCP implementation using ICPs	One day training of ICPs and IPCP implementation	IPCP quiz JEFFSATIC PINCOM-Q Audio-visual recording	Participant responses	All health professionals, representative from the municipality health office, co-researchers and consultant who contribute in designing the model
		Medical record review Observation	Modified McMaster Ottawa Scale	Collaborative Communication Share leadership conflict resolution Team functioning Role and responsibility	All health professionals who use the ICPs
**Evaluating stage**					
Evaluation and improvement of IPCPs implementation	ICPs refinement	In-depth interview	Audio recording. Interview and FGD guideline	Implementation outcome: Acceptability Appropriateness Adoption Feasibility Implementation cost	All health professionals, co-researchers and patient/family who employ the ICP in patient management
		FGDs			

### Trustworthiness

Natural situations should be examined in qualitative studies to ensure natural, evidence-based, prolonged engagement that has built trust and rapport in the participants [74]. The first author is the principal investigator of this study and a female physician who has been working at the tertiary hospital where the study will be conducted. She visits the primary and secondary care facilities on a regular basis and is therefore familiar with all the health professionals working at these facilities. Prior to data collection activities, all participants will be approached through face to face interaction in their workplace. Participants were aware that this research is part of her dissertation project to improve maternal services for pregnant women with cardiac diseases through applying ICPs implementation. All participants have appropriate understanding of the study purpose and their roles prior to engagement in this study.

ICPs will be implemented in this study through training, ongoing monitoring and consultation. Therefore, it is likely that others may be present besides the participants and researcher. For example the patient’s family and administration staff. At the beginning of this research, in-depth interviews, FGDs and document analysis will be conducted to summarize the results of the context and situation analysis. Data collected in each stage will be shared with previous and subsequent participants to encourage full participation. ICPs and IPCP training will be held to promote the involvement of all participants in the ICPs implementation [75]. Data from the medical record reviews will be triangulated with observations, in-depth interviews and FGDs results. All interviews and FGDs will be audio-recorded and transcripts will be confirmed by informants through peer debriefing and member checking. A thick description will be produced to provide behavioural, cultural and contextual findings, thus allowing the research to be readily replicated [74].

## Discussion

IPCP has been hypothesized to improve health outcome through enhancing interprofessional communication, resources utilization efficiency and patient safety [27, 76]. This study will provide comprehensive information about the benefits of, success and barrier factors of IPCP for pregnant woman with heart diseases referral care. This study implements and evaluates the IPCP in the primary, secondary to tertiary health care facilities and develops ICPs as a tool to enhance IPCP through defining a clear role and responsibilities of each health professionals based on their workplace. Perception, attitude, and experience of both health professionals and patients will be determined during the process.

To our knowledge, this will be the first study on IPCP involving multi-level maternal health services in Indonesia. It is expected to produce significant evidence on the improvement of IPCP on maternal referral care for pregnancy with heart diseases, the third leading indirect cause of maternal mortality [77]. The PAR study design will engage health professionals in all four stages (need assessment, planning, implementation, and evaluation) [78]. It will improve IPCP understanding, which will lead to better professional habits, mindset changes, and discipline to effectively implement IPCP [79]. Findings from this study will inform health service planner and policymaker to strengthen the practice of IPCP during the referral process. Thus, its implementation is expected to provide high-quality care for pregnant women with heart diseases as the long term impact.

The study may face some potential methodological and practical challenges. The study setting is limited to one tertiary hospital and its referral network, the patient population and the service within which we are seeking to implement this pathway. This could limit the generalizability of findings to other hospitals with different characteristics. Further adaptations may be required to implement similar efforts in Regional public hospitals. In terms of PAR, participation is the key to success. We are aware that time constraints and workload of each participant may vary, but we are confident that intense communication during training, monitoring and continuous discussions could improve participants’ contribution in this study [80–82]. Concerning potential biases due to the role of the first author in the hospitals, her familiarity and long experiences working in the study setting may be useful to deepen the understanding gained from the interview/FGDs.

## Data Availability

The datasets generated and analysed during the current study will be included in the subsequent publication of the results.

## Abbreviations

BEmNOC: Basic Emergency Obstetric Care
CEmNOC: Comprehensive Emergency Obstetric Care
COREQ: COnsolidated criteria for REporting Qualitative research
DRP: Demak Regional Public
FGD: Focus Group Discussion
ICD: International Classification of Diseases
ICPs: Integrated Care Pathways
ICU: Intersive Care Unit
IPCP: Interprofessional Collaboration Practice
MMR: Maternal Mortality Rate
PAR: Participatory Action Research
PHC: Public Health Centre
PI: Principal Investigator
PIC: Person in charge
RA: Research Assistant
SAI: Sultan Agung Islamic
